# Biological enrichment prediction of polychlorinated biphenyls and novel molecular design based on 3D-QSAR/HQSAR associated with molecule docking

**DOI:** 10.1042/BSR20180409

**Published:** 2019-05-17

**Authors:** Jiawen Yang, Wenwen Gu, Yu Li

**Affiliations:** 1 College of Environmental Science and Engineering, North China Electric Power University, Beijing, China; 2 Moe Key Laboratory of Resources and Environmental Systems Optimization, North China Electric Power University, Beijing, China

**Keywords:** biological enrichment, 3D-QSAR, HQSAR, PCBs, molecular modification, molecular docking

## Abstract

Based on the experimental data of octanol-water partition coefficients (*K*_ow_, represents bioaccumulation) for 13 polychlorinated biphenyl (PCB) congeners, comparative molecular field analysis (CoMFA) and comparative molecular similarity indices analysis (CoMSIA) were used to establish 3D-QSAR models, combined with the hologram quantitative structure–activity relationship (HQSAR), the substitution sites (mono-substituted and bis-substituted) and substituent groups (electron-withdrawing hydrophobic groups) that significantly affect the octanol-water partition coefficients values of PCBs were identified, a total of 63 monosubstituted and bis-substituted were identified. Compared with using 3D-QSAR model alone, the coupling of 3D-QSAR and HQSAR models greatly increased the number of newly designed bis-substituted molecules, and the log*K*_ow_ reduction in newly designed bis-substituted molecules was larger than that of monosubstituted molecules. This was established to predict the *K*_ow_ values of 196 additional PCBs and carry out a modification of target molecular PCB-207 to lower its *K*_ow_ (biological enrichment) significantly, simultaneously maintaining the flame retardancy and insulativity after calculation by using Gaussian09. Simultaneously, molecular docking could further screen out three more environmental friendly low biological enrichment newly designed PCB-207 molecules (5-methyl-PCB-207, 5-amino-PCB-207, and 4-amino-5-ethyl-PCB-207).

## Introduction

Polychlorinated biphenyls (PCBs for short) are the general names of compounds based on the substitution of a hydrogen atom on a benzene ring with a chlorine atom, including 209 congeners characterized by the number and position of the chlorine atoms on the biphenyl core. PCBs are one of the persistent organic pollutants (POPs); PCBs are high in stability, toxicity, environmental persistence, bioaccumulation, long-distance migration ability, and other characteristics. PCBs have the advantages of low solubility, high dielectric constant, low vapor pressure, good heat resistance, and excellent insulating properties. PCBs widely served as flame retardant, paint, dielectric fluids in capacitors, insulating oil in transformers, and as a plasticizer agent and rubber sealants [1].

In the 1970s, PCBs were prohibited from being produced and used worldwide [2]. However, because of the high stability of PCBs and PCBs themselves being resistant to their natural degradation process, the discontinuation does not mean that the hidden dangers are eliminated, and the destructiveness of PCBs can persist for several years. Today, 40 years after the PCBs have been banned globally, PCB residues are still universally detected in organisms and various environmental media in the world, affecting various organisms including humans through the cumulative effect of the food chain. The hazards to environment posed by PCBs are longer term and more complex and have become global environmental issues that pose a serious threat to human health and the ecological environment. Therefore, studies on PCBs are of great significance in the environmental pollution and biological health aspects and have received substantially more global attention.

3D-QSAR, which could reveal the structure–activity relationship of compounds by analyzing the changes of 3D spatial fields around the superposed molecules, overcomes the limitations of the conventional 2D model in characterizing the relationship between property and substance and have a clearer physical meaning and more abundant information of the molecular field energy, thus 3D-QSAR is more widely used in environmental science and toxicology and other fields than 2D-QSAR [3]. Some studies have also used some kind of bacteria to establish the method of toxicity test of some chemicals, and obtained good research results [4,5]. Su et al. [6] determined the combined toxicity of phenolic compounds and heavy metal lead to the photobacterium and then conducted a QSAR study. In addition to the use of 3D-QSAR method, this dissertation also combined hologram quantitative structure–activity relat

ionship (HQSAR) method together to explore the mechanisms that affect the activity of PCB compounds.

In recent years, various methods have been used to estimate the biological enrichment coefficients of PCBs in various mediums, and to assess the biological enrichment degree of PCBs in sediments and various biological mediums. The concentration of PCBs detected in main species in the food chain of the Antarctic and Arctic has reached moderate levels of contamination [7]. Engel et al. [8] used logistic regression analysis to find that some PCBs in serum were associated with an increased risk of lymphoma. Experts in environmental field at home and abroad have also conducted a great deal of research on the migration and conversion behavior, ecological effects, toxicity as well as pollution elimination methods of PCBs in water bodies, sediments, soils, and organisms [9–14], including microbial degradation and bioremediation methods of PCBs [15]. At present, the researches on PCBs in terms of toxicology, exposure characteristics, adsorption, and degradation have been relatively comprehensive, but there is a lack of research on the modification of the POPs characteristics at the molecular structure level.

Although PCBs have been banned, but there are still residues on the global scale, and the modification of PCBs in the present paper was based on the residual PCBs. PCBs have the advantages of various commercial functional properties, and modification of PCB molecules makes sense if the POP properties of newly designed PCB molecules are reduced or even removed from POPs without changing their functional properties.

In the present paper, HQSAR and 3D-QSAR models were established by using with 13 PCB octanol-water partition coefficients (*K*_ow_) from Hawker and Connell [16]. The substitution sites and substituent groups that affect the octanol-water partition coefficients of PCBs were determined by combining these two models. And carry out a modification of target molecular PCB-207 to lower its *K*_ow_ (biological enrichment). Only using the 3D contour plot of 3D-QSAR to determine the substitution sites and substituent groups, the fewer sites and groups can be obtained, and almost impossible to do the double-site substitution. Less scheme on molecular modification, it is not enough to provide reference for future research. The octanol-water partition coefficient (*K*_ow_), which can reflect the distribution ability of organic compounds between octanol and aqueous phases, is one of the effective index of the distribution of organic compound in environmental media (water, soil, and sediment) and is also one of the important property parameters used to study the environmental behavior of organic compounds. Additionally, *K*_ow_ can simulate the distribution of organic between biological phase and water phase, and it is closely related to the toxicity [17], biological enrichment [18], and solubility [19] of compounds.

Through the evaluation of toxicity, persistence, and long-range mobility, the low biological enrichment newly designed PCB-207 molecules, all the POP characteristic parameters are reduced while the actual functional properties are not changed, fulfilling their industrial and commercial functional requirements and greatly reducing the environmental impact. Furthermore, the correlation analysis between the number of Cl atoms in PCBs and the average value of log*K*_ow_ of isomer in chlorobiphenyl to decachlorobiphenyl, respectively, showed that with the increase in the number of Cl atoms, the log*K*_ow_ values also increase and the biological enrichment capability becomes stronger, and the results of molecular docking between PCBs and degrading enzyme BphA also confirmed this conclusion.

The target pollutant PCB-207 selected in the present paper has large observed log*K*_ow_ value of 7.52. After modification, the log*K*_ow_ values of newly designed molecules decreased and the lowest reached 5.489. When log*K*_ow_ <5, the compounds were judged to be divorced from POPs, if the modification is carried out by using PCBs with less chlorine atoms as target molecular, which the log*K*_ow_ values of the compounds are relatively small, the log*K*_ow_ values can also be reduced by the same extent, then that of modified compounds must be less than 5, which can make the newly designed compounds out of the scope of POPs. In the present paper, we mainly come up with a modified method for the residual PCBs range from chlorobiphenyl to decachlorobiphenyl, which provides a theoretical basis in methods for the conversion of residual PCBs into low environmental impact compounds. In addition, the molecular docking technique was used to further study the biological enrichment of the modified molecules on the liver tissue where the liver enzymes exist in, and it was found that not all of bioaccumulation of newly designed molecules of low biological enrichment of PCB-207 was reduced on liver tissue. But it can be screened out that the several kinds of low biological enrichment newly designed PCB-207 molecules from all newly designed PCB-207 molecules have lower biological enrichment in liver tissue where most liver enzymes exist in, and provide an important theoretical method for the study of bioaccumulation of PCBs in the future. The present paper is expected to provide a theoretical foundation for further study of biological enrichment of PCBs.

## Materials and methods

### The establishment of PCBs bioconcentration 3D-QSAR/HOSAR models

The comparative molecular field analysis (CoMFA), comparative molecular similarity indices analysis (CoMSIA) models, HQSAR model that was used for *K*_ow_ value prediction and low biological enrichment molecular design were established with Sybyl-X 2.0 software to analyze the 3D/HQSAR models and perform the molecular docking.

The lowest energy conformation of the molecule was used as the advantage stable conformation; the geometry of these compounds was subsequently optimized using Tripos force field with the Gasteiger-Huckel charges. Repeated minimizations were performed by the Powell method with a maximum iteration of 10000 to reach an energy convergence gradient value of 0.005 kJ/mol, and others are default values.

To establish the 3D-QSAR models, the whole dataset (containing 13 compounds) was divided 3:1 into a training set (containing ten compounds) for 3D-QSAR model generation and a test set (containing three compounds) for model validation for its accuracy and stability. Compounds in the training set were selected based on their ability to appropriately represent the structural diversity of the whole dataset and cover the range of log*K*_ow_ values [20,21]. In this paper, we have chosen a set of training set and test set that both internal and external verifications were qualified to establish 3D-QSAR model. In this process, the 2,2′,3,3′,4,4′,5,5′-octachlorobiphenyl (PCB-194) molecule with the largest log*K*_ow_ was used as the template and the benzene ring skeleton was used as a model skeleton to align the other compounds using the Align Database command in Sybyl. The 3D-QSAR of CoMFA and CoMSIA was directly yielded by partial least squares (PLS) analyses in which the ten log*K*_ow_ values of PCBs in training set served as the dependent variable, and the 3D structure of ten PCBs served as the independent variable. In this process, the leave-one-out (LOO) cross-validation procedure was performed to determine the optimum number of components (*n*) and the highest cross-validation correlation coefficient (q^2^) for the correlation models. Simultaneously, noncross-validated analysis was performed. The quality of the models was measured by cross-validation correlation coefficient (q^2^), noncross-validation correlation coefficient (r^2^), standard error of estimate (SEE), and Fisher test (F) values. Finally, the relationship between the log*K*_ow_ of the PCB compounds and each field was expressed as a 3D contour plot.

HQSAR model does not require the selection of active conformations and the alignment of PCB molecules, HQSAR and PLS analysis was conducted using the Sybyl-X 2.0 package by Tripos Company. HQSAR calculations were carried out using three distinct parameters the fragment distinction, the fragment size, and the hologram length. Use SYBYL-HQSAR module to generate molecular hologram. The HQSAR calculation provides 12 default prime number (53, 59, 61, 71, 83, 97, 151, 199, 257, 307, 353, and 401) molecular hologram lengths by default. The HQSAR model can be optimized by changing the fragment distinction and the molecular fragment size. The fragment discrimination represents the topology parameter mapped in the molecular holography, which included atoms (A), bonds (B), connections (C), hydrogen atoms (H), chirality (Ch), and donor and acceptor (DA), in which atoms (A) can distinguish the different types of atoms; the bonds (B) can identify the difference amongst the chemical bonds formed by the atoms; connections (C) can reveal the hybridization state of atoms inside the fragment; Ch can obtain stereochemical information of atomic Ch and chemical bonds in fragments; hydrogen bond DA can ascertain hydrogen bond donor or acceptor of fragments. In general, the default setting A/B/C for the fragment distinction has already contained the basic information required to distinguish different fragments. On this basis, adding other fragment distinction parameters, establishing different combinations of fragment distinction parameters, selecting different fragment sizes. And the HQSAR models were obtained in specific preset parameters. The fragment size is the number of atoms contained in the fragment: (1–3) means the smaller atomic fragments, which can characterize the atomic type and functional groups, approximately (4–7) means medium atomic fragments that could distinguish the chain length of the alicyclic hydrocarbon or the characters and the structural features such as the substitution sites and substituent groups of the aromatic hydrocarbon; (8–10) means larger atomic fragments.

HQSAR models (method) were conducted according to the different parameter settings, including the hologram length (HL) values (53, 59, 61, 71, 83, 97, 151, 199, 257, 307, 353, and 401), and the fragment size (4–7) by default. Based on the default setting A/B/C of the fragment distinction, eight kinds of fragment distinction combinations are constituted after adding other fragment distinction parameters, and the combination modes are A/B/C, A/B/C/H, A/B/C/Ch, A/B/C/DA, A/B/C/H/Ch, A/B/C/H/DA, A/B/C/Ch/DA, and A/B/C/H/Ch/DA. Molecular hologram of 13 training set molecules and activity were used as the independent and dependent variables, respectively. A series of HQSAR models were obtained by taking linear regression analysis through PLS. Then, the prediction ability, stability, and fitting capacity of these models were validated by a high cross-validated correlation coefficient q^2^, noncross-validated correlation coefficient r^2^ values, and a low-SEE along with a low cross-validated SEE (SE_cv_). The HQSAR analysis results can be graphically displayed in the form of contribution maps, in which the color coding of each atom reflects its contribution to the activity of the whole molecule. Hence, we can obtain the favorable information of molecular modification [22].

The substitution sites identified by HQSAR models that have a significant effect on the biological enrichment activity of the target molecular, combined with the distribution characteristics of the force field (hydrophobic field and hydrophobic group) that has a significant effect on the bioaccumulation of the target molecular determined by CoMSIA model of 3D-QSAR. It can precisely locate the low biological enrichment activity modified substitution sites and the substituent groups of target molecular, and then modify the target molecules.

### Molecular docking of PCBs with enzymes before and after molecular modification

In the present paper, the molecular docking was performed by using SYBYL-X 2.0 software from Tripos Company in the United States. The molecular docking of PCB-207/newly designed PCB-207 that have low biological enrichement with target protein of liver enzymes were both the docking that between small molecular and protein. Therefore, use Surflex-dock module by SYBYL software for the semiflexible docking [23,24]. Open the Surflex-dock module to correct and modify the protein that is about to carry on molecular docking, and extract the ligand from it to determine the binding sites. Set up docking pockets for the prepared protein, put the ligand molecules that are about to carry on molecular docking into the docking pocket, prepare for the molecular docking to take place. The evaluation standards of molecular docking results include Crash, Polar, and Total Score three expression functions. Crash represents the inadaptation of docking for the ligand into the receptor, the closer to 0 the better, that is the smaller the absolute value the better; Polar stands for the polarity function score, which the higher the scores the better when the binding molecule sites on the binding surface while the lower the scores the better when the binding site is inside the molecule; Total Score denotes the comprehensive score of above parameters, the higher the score the better. According to Total Score, it could be to determine the appropriateness of docking between molecules and proteins.

### Calculation methods of newly designed PCB molecules’ functional properties

Quantum chemical descriptor of PCB-207 molecules and low biological enrichment newly designed PCB-207 molecules were calculated using Gaussian09, including energy gap (insulativity parameter) and C–Cl bond dissociation enthalpy (flame retardancy parameter). The geometry of all compounds was optimized using B3LYP/6-31g (d, p) level of density functional theory [25].

The calculation orbit (highest occupied molecular orbital (HOMO) and lowest unoccupied molecular orbital (LUMO)), which is significant to the study of molecular physics and chemical properties, is an important parameter of quantum chemistry. The energy gap, which is the energy difference of the HOMO and LUMO, is the required lowest energy of the electron excitation process, and is also an important parameter of conductivity and luminescence [25]. In addition, the larger the energy gap, the weaker the conductivity. The insulation information of newly designed molecules can be obtained by calculating the energy gap value.

By comparing the C–Cl bond dissociation enthalpy of PCB-207 molecules and low biological enrichment newly designed PCB-207 molecules, we obtained the flame retardancy information about low biological enrichment newly designed PCB-207 molecules. The lower the C–Cl bond dissociation enthalpy, indicating that the newly designed PCB-207 molecules after modification are more likely to release Cl-free radicals and HCl for inflaming retarding.

## Results and analysis

### Analysis, evaluation, and verification of PCBs molecular bioaccumulation based on CoMFA and CoMSIA model

The CoMFA in the 3D-QSAR model mainly reflects the non-bonding interaction between the drug molecules and the receptor, and is a widely used indirect drug design method. The steric fields’ energy and electrostatic fields’ energy are calculated using the Lennard-Jones and Coul potential energy functions. However, the lattice points of molecular surfaces are ignored due to the rapid increase in van der Waals repulsion. Compared with the CoMFA method, the CoMSIA method uses Gaussian similarity functions, which avoids drastic changes of the potential energy at the lattice points on the molecular surface, improves the sensitivity to the molecular alignment method and the spatial orientation, and increases hydrophobic fields in addition to hydrogen bond-donor and hydrogen bond-acceptor fields. The results of the CoMSIA method show a relatively small influence from compound matching rules and can more intuitively explain the quantitative structure–activity relationship of a compound. The use of CoMSIA can overcome inherent defects of CoMFA but does not necessarily gain better results [26]. Therefore, in this study, two methods (CoMFA and CoMSIA) were used to verify and supplement each other to gain reliable predicting models.

The statistical parameters of the CoMFA model and the CoMSIA model were shown in Table 1. According to Table 1, the CoMFA model and the CoMSIA model both had an optimum *n* of 2, the cross-validated q^2^ of 0.784, 0.883 (>0.5), the SEE of 0.215, 0.177, the noncross-validated r^2^ of 0.954, 0.969 (>0.8), the F-value of 72.595, 108.026, and the scrambling stability test parameters Q^2^ of 0.715, 0.787, cSDEP of 0.525, 0.452, and dQ^2^/dr^2^yy of 1.625, 1.309, respectively, illustrating that these two models had both suitable fitting predictive abilities [27,28].

**Table 1:** Statistical parameters and molecular field’s contribution to PCBs *K*_ow_ of the CoMFA and CoMSIA models

Model	q^2^	*n*	SEE	r^2^	F	r^2^_pred_	SEP	Q^2^	Q^2^_ext_	cSDEP	dq^2^/dr^2^yy	S	E	H	D	A
CoMFA	0.784	2	0.215	0.954	72.595	0.896	0.301	0.715	0.996	0.525	1.625	33.90%	66.10%	-	-	-
CoMSIA	0.883	2	0.177	0.969	108.026	0.937	0.234	0.787	0.891	0.452	1.309	0.90%	84.40%	14.70%	0	0

Abbreviations: A, hydrogen bond-acceptor; cSDEP, calculated cross-validated standard error of prediction; D, hydrogen bond-donor; E, electrostatic; F, Fisher test value; H, hydrophobic; *n*, optimum number of component; q^2^, cross-validated correlation coefficient after the LOO procedure; Q^2^_ext_, the explained variance in prediction; r^2^, noncross-validated correlation coefficient; r^2^_pred_, correlation coefficient for test set predictions; S, steric; SEP, standard error of prediction.

As is shown in Table 1, the corresponding contribution percentages of the steric (S) and electrostatic (E) fields to the CoMFA model were 33.90 and 66.10%, respectively, indicating that the steric and electrostatic distributions of the groups may influence the *K*_ow_ values of the PCB homologs. Verifying electrostatic interaction was the major contribution to the log*K*_ow_ of PCBs. The CoMSIA defines explicit hydrophobic and hydrogen-bond DA descriptors in addition to the steric and electrostatic fields in CoMFA. The contributions of the steric (S), electrostatic (E), hydrophobic (H), hydrogen bond-donor (D), and hydrogen bond-acceptor (A) fields to the CoMSIA model were 0.90, 84.40, 14.70, 0.0, and 0.00%, respectively.

Take above discussion into consideration, the steric distributions, electrostatic distributions, and hydrophobic properties of the groups may influence the *K*_ow_ values of the PCBs homologs, the electrostatic distribution had the most influence, and the hydrogen bond-donor and hydrogen bond-acceptor fields had no effect.

**Figure 1:**
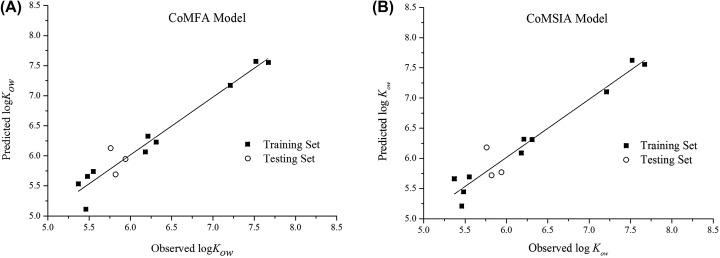
Plot of observed compared with predicted log*K*_ow_ values using the CoMFA model (**A**) and CoMSIA model (**B**). Plot of observed compared with predicted log*K*_ow_ values using the CoMFA model (**A**) and CoMSIA model (**B**).

External validation was also conducted to further assess the reliabilities and the predictive ability of the built models. The test set with three compounds was used for this validation. The r^2^_pred_ of 0.896 (>0.6), 0.937 (>0.6), the SEP of 0.301, 0.234, the Q^2^_ext_ of 0.996 (>0.5), 0.891 (>0.5) were achieved, verifying the good external predictive ability of the two models [28]. The correlations between the experimental log*K*_ow_ values and log*K*_ow_ values predicted by the CoMFA and CoMSIA models are depicted in Figure 1A,B, respectively. In further analysis of the scatter plot of experimental versus log*K*_ow_ values predicted by the CoMFA and CoMSIA models revealed a fine linear dependence (*r^2^* of 0.9579 and 0.9641, respectively) among experimental values and predicted values, all data were concentrated around the trend line. And the slopes of the linear equations for experimental and predicted values were 0.96105 and 0.96343, respectively, which can be observed from the scatter plot of experimental versus predicted values of log*K*_ow_, which can be observed from the scatter plot of experimental versus predicted values of log*K*_ow_, which indicates the two models have good internal prediction ability and can be used for the prediction of PCBs *K*_ow_ values.

### Prediction of PCBs log*K*_ow_ based on CoMFA and CoMSIA models

CoMFA and CoMSIA models were used to predict 209 types of PCBs, and the experimental and predicted log*K*_ow_ values and standard deviations for the PCBs are given in Table 2.

**Table 2:** Predicted log*K*_ow_ values of PCBs through CoMFA and CoMSIA models

Number	Compounds	Obs.	CoMFA		CoMSIA	
			Pred.	Relative error (%)	Pred.	Relative error(%)
1	2-Chlorobiphenyl		5.443		5.097	
2	3-Chlorobiphenyl		4.921		4.804	
3	4-Chlorobiphenyl		5.130		4.962	
4	2,2′-Dichlorobiphenyl		5.175		4.897	
5	2,3-Dichlorobiphenyl		5.385		5.215	
6	2,3′-Dichlorobiphenyl		5.444		5.357	
7	2,4-Dichlorobiphenyl		5.296		5.310	
8	2,4′-Dichlorobiphenyl		5.335		5.256	
9	2,5-Dichlorobiphenyl		5.344		5.217	
10	2,6-Dichlorobiphenyl		5.054		4.890	
11	3,3′-Dichlorobiphenyl		5.540		5.363	
12	3,4-Dichlorobiphenyl		5.807		5.549	
13	3,4′-Dichlorobiphenyl		5.506		5.469	
14	3,5-Dichlorobiphenyl		5.658		5.505	
15	4,4′-Dichlorobiphenyl		5.818		5.603	
16	2,2′,3-Trichlorobiphenyl		5.610		5.320	
17	2,2′,4-Trichlorobiphenyl		5.602		5.349	
18	2,2′,5-Trichlorobiphenyl		5.640		5.310	
19	2,2′,6-Trichlorobiphenyl		5.548		5.166	
20	2,3,3′-Trichlorobiphenyl		6.342		5.730	
21	2,3,4-Trichlorobiphenyl		5.942		5.770	
22	2,3,4′-Trichlorobiphenyl		5.799		5.667	
23	2,3,5-Trichlorobiphenyl		5.974		5.653	
24	2,3,6-Trichlorobiphenyl		5.816		5.507	
25	2,3′,4-Trichlorobiphenyl		5.897		5.633	
26	2,3′,5-Trichlorobiphenyl		5.612		5.500	
27	2,3′,6-Trichlorobiphenyl		5.183		5.258	
28	2,4,4′-Trichlorobiphenyl		5.710		5.761	
29	2,4,5-Trichlorobiphenyl		5.714		5.721	
30	2,4,6-Trichlorobiphenyl		5.470		5.342	
31	2,4′,5-Trichlorobiphenyl		5.759		5.669	
32	2,4′,6-Trichlorobiphenyl		5.429		5.395	
33	2,3′,4′-Trichlorobiphenyl		5.854		5.805	
34	2,3′,5′-Trichlorobiphenyl		5.571		5.676	
35	3,3′,4-Trichlorobiphenyl		5.913		5.867	
36	3,3′,5-Trichlorobiphenyl		5.947		5.771	
37	3,4,4′-Trichlorobiphenyl		6.224		6.001	
38	3,4,5-Trichlorobiphenyl		6.068		5.952	
39	3,4′,5-Trichlorobiphenyl		6.034		6.012	
40^1^	2,2′,3,3′-Tetrachlorobiphenyl	5.55	5.738	3.39%	5.693	2.58%
41	2,2′,3,4-Tetrachlorobiphenyl		5.992		5.823	
42	2,2′,3,4′-Tetrachlorobiphenyl		6.037		5.772	
43	2,2′,3,5-Tetrachlorobiphenyl		6.078		5.733	
44	2,2′,3,5′-Tetrachlorobiphenyl		6.154		5.880	
45	2,2′,3,6-Tetrachlorobiphenyl		5.515		5.492	
46	2,2′,3,6′-Tetrachlorobiphenyl		5.644		5.460	
47	2,2′,4,4′-Tetrachlorobiphenyl		5.989		5.854	
48	2,2′,4,5-Tetrachlorobiphenyl		6.141		5.906	
49	2,2′,4,5′-Tetrachlorobiphenyl		6.067		5.762	
50	2,2′,4,6-Tetrachlorobiphenyl		5.452		5.572	
51	2,2′,4,6′-Tetrachlorobiphenyl		5.491		5.519	
52	2,2′,5,5′-Tetrachlorobiphenyl		6.185		5.870	
53^1^	2,2′,5,6′-Tetrachlorobiphenyl	5.46	5.656	3.59%	5.446	0.26%
54^1^	2,2′,6,6′-Tetrachlorobiphenyl	5.48	5.114	6.68%	5.209	4.95%
55	2,3,3′,4-Tetrachlorobiphenyl		6.399		6.182	
56	2,3,3′,4′-Tetrachlorobiphenyl		6.319		6.216	
57	2,3,3′,5-Tetrachlorobiphenyl		6.335		6.181	
58	2,3,3′,5′-Tetrachlorobiphenyl		6.035		6.087	
59	2,3,3′,6-Tetrachlorobiphenyl		5.606		5.677	
60	2,3,4,4′-Tetrachlorobiphenyl		6.316		6.276	
61^1^	2,3,4,5-Tetrachlorobiphenyl	6.18	6.067	1.83%	6.087	1.50%
62	2,3,4,6-Tetrachlorobiphenyl		5.995		5.895	
63	2,3,4′,5-Tetrachlorobiphenyl		6.226		6.081	
64	2,3,4′,6-Tetrachlorobiphenyl		5.959		5.951	
65^2^	2,3,5,6-Tetrachlorobiphenyl	5.94	5.947	0.12%	5.770	2.86%
66^1^	2,3′,4,4′-Tetrachlorobiphenyl	6.31	6.229	1.28%	6.311	0.02%
67	2,3′,4,5-Tetrachlorobiphenyl		6.237		6.273	
68	2,3′,4,5′-Tetrachlorobiphenyl		5.945		6.182	
69	2,3′,4,6-Tetrachlorobiphenyl		6.296		6.239	
70	2,3′,4′,5-Tetrachlorobiphenyl		6.278		6.219	
71	2,3′,4′,6-Tetrachlorobiphenyl		5.852		5.813	
72	2,3′,5,5′-Tetrachlorobiphenyl		5.994		6.089	
73	2,3′,5′,6-Tetrachlorobiphenyl		5.938		5.723	
74	2,4,4′,5-Tetrachlorobiphenyl		6.130		6.172	
75	2,4,4′,6-Tetrachlorobiphenyl		5.844		5.847	
76	2,3′,4′,5′-Tetrachlorobiphenyl		5.981		6.122	
77^1^	3,3′,4,4′-Tetrachlorobiphenyl	6.21	6.327	1.88%	6.317	1.72%
78	3,3′,4,5-Tetrachlorobiphenyl		6.479		6.353	
79	3,3′,4,5′-Tetrachlorobiphenyl		6.440		6.409	
80	3,3′,5,5′-Tetrachlorobiphenyl		6.475		6.313	
81	3,4,4′,5-Tetrachlorobiphenyl		6.444		6.458	
82	2,2′,3,3′,4-Pentachlorobiphenyl		6.120		6.141	
83	2,2′,3,3′,5-Pentachlorobiphenyl		6.206		6.051	
84	2,2′,3,3′,6-Pentachlorobiphenyl		5.639		5.811	
85	2,2′,3,4,4′-Pentachlorobiphenyl		6.419		6.274	
86	2,2′,3,4,5-Pentachlorobiphenyl		6.458		6.233	
87	2,2′,3,4,5′-Pentachlorobiphenyl		6.537		6.382	
88	2,2′,3,4,6-Pentachlorobiphenyl		6.894		5.995	
89	2,2′,3,4,6′-Pentachlorobiphenyl		6.323		6.158	
90	2,2′,3,4′,5-Pentachlorobiphenyl		6.505		6.185	
91	2,2′,3,4′,6-Pentachlorobiphenyl		6.939		5.944	
92	2,2′,3,5,5′-Pentachlorobiphenyl		6.632		6.293	
93	2,2′,3,5,6-Pentachlorobiphenyl		5.985		5.904	
94	2,2′,3,5,6′-Pentachlorobiphenyl		6.033		6.077	
95	2,2′,3,5′,6-Pentachlorobiphenyl		6.063		6.053	
96	2,2′,3,6,6′-Pentachlorobiphenyl		5.574		5.620	
97	2,2′,3,4′,5′-Pentachlorobiphenyl		6.577		6.329	
98	2,2′,3,4′,6′-Pentachlorobiphenyl		6.066		5.912	
99	2,2′,4,4′,5-Pentachlorobiphenyl		6.528		6.411	
100	2,2′,4,4′,6-Pentachlorobiphenyl		5.877		6.024	
101	2,2′,4,5,5′-Pentachlorobiphenyl		6.607		6.319	
102	2,2′,4,5,6′-Pentachlorobiphenyl		6.220		6.113	
103	2,2′,4,5′,6-Pentachlorobiphenyl		6.078		5.898	
104^1^	2,2′,4,6,6′-Pentachlorobiphenyl	5.37	5.532	3.02%	5.661	5.42%
105^2^	2,3,3′,4,4′-Pentachlorobiphenyl	5.82	5.692	2.20%	5.720	1.72%
106	2,3,3′,4,5-Pentachlorobiphenyl		6.525		6.499	
107	2,3,3′,4′,5-Pentachlorobiphenyl		6.746		6.630	
108	2,3,3′,4,5′-Pentachlorobiphenyl		6.827		5.596	
109	2,3,3′,4,6-Pentachlorobiphenyl		5.978		6.180	
110	2,3,3′,4′,6-Pentachlorobiphenyl		5.980		6.132	
111	2,3,3′,5,5′-Pentachlorobiphenyl		6.462		6.500	
112	2,3,3′,5,6-Pentachlorobiphenyl		6.070		6.089	
113	2,3,3′,5′,6-Pentachlorobiphenyl		6.470		6.279	
114	2,3,4,4′,5-Pentachlorobiphenyl		6.441		5.593	
115	2,3,4,4′,6-Pentachlorobiphenyl		6.370		6.400	
116	2,3,4,5,6-Pentachlorobiphenyl		6.123		6.212	
117	2,3,4′,5,6-Pentachlorobiphenyl		6.366		6.222	
118	2,3′,4,4′,5-Pentachlorobiphenyl		6.648		6.722	
119	2,3′,4,4′,6-Pentachlorobiphenyl		6.355		6.628	
120	2,3′,4,5,5′-Pentachlorobiphenyl		6.365		6.592	
121	2,3′,4,5′,6-Pentachlorobiphenyl		6.356		6.175	
122	2,3,3′,4′,5′-Pentachlorobiphenyl		6.445		6.533	
123	2,3′,4,4′,5′-Pentachlorobiphenyl		6.355		6.628	
124	2,3,4′,5,5′-Pentachlorobiphenyl		6.454		6.558	
125	2,3′,4′,5′,6-Pentachlorobiphenyl		6.127		6.261	
126	3,3′,4,4′,5-Pentachlorobiphenyl		6.851		6.856	
127	3,3′,4,5,5′-Pentachlorobiphenyl		6.888		6.759	
128	2,2′,3,3′,4,4′-Hexachlorobiphenyl		6.548		6.591	
129	2,2′,3,3′,4,5-Hexachlorobiphenyl		6.706		6.646	
130	2,2′,3,3′,4,5′-Hexachlorobiphenyl		6.664		6.701	
131	2,2′,3,3′,4,6-Hexachlorobiphenyl		6.018		6.313	
132	2,2′,3,3′,4,6′-Hexachlorobiphenyl		6.138		6.280	
133	2,2′,3,3′,5,5′-Hexachlorobiphenyl		6.750		6.611	
134	2,2′,3,3′,5,6-Hexachlorobiphenyl		6.125		6.223	
135	2,2′,3,3′,5,6′-Hexachlorobiphenyl		6.187		6.371	
136^2^	2,2′,3,3′,6,6′-Hexachlorobiphenyl	5.76	6.129	6.41%	6.181	7.31%
137	2,2′,3,4,4′,5-Hexachlorobiphenyl		6.885		6.685	
138	2,2′,3,4,4′,5′-Hexachlorobiphenyl		6.959		6.831	
139	2,2′,3,4,4′,6-Hexachlorobiphenyl		6.318		6.446	
140	2,2′,3,4,4′,6′-Hexachlorobiphenyl		6.439		6.414	
141	2,2′,3,4,5,5′-Hexachlorobiphenyl		7.003		6.793	
142	2,2′,3,4,5,6-Hexachlorobiphenyl		6.362		6.404	
143	2,2′,3,4,5,6′-Hexachlorobiphenyl		6.470		6.372	
144	2,2′,3,4,5′,6-Hexachlorobiphenyl		6.441		6.555	
145	2,2′,3,4,6,6′-Hexachlorobiphenyl		6.075		6.219	
146	2,2′,3,4′,5,5′-Hexachlorobiphenyl		7.046		6.742	
147	2,2′,3,4′,5,6-Hexachlorobiphenyl		6.409		6.356	
148	2,2′,3,4′,5,6′-Hexachlorobiphenyl		6.522		6.325	
149	2,2′,3,4′,5′,6-Hexachlorobiphenyl		6.481		6.502	
150	2,2′,3,4′,6,6′-Hexachlorobiphenyl		5.991		6.072	
151	2,2′,3,5,5′,6-Hexachlorobiphenyl		6.534		6.465	
152	2,2′,3,5,6,6′-Hexachlorobiphenyl		6.012		6.049	
153	2,2′,4,4′,5,5′-Hexachlorobiphenyl		6.992		6.821	
154	2,2′,4,4′,5,6′-Hexachlorobiphenyl		6.330		6.427	
155	2,2′,4,4′,6,6′-Hexachlorobiphenyl		6.908		6.166	
156	2,3,3′,4,4′,5-Hexachlorobiphenyl		7.115		7.131	
157	2,3,3′,4,4′,5′-Hexachlorobiphenyl		7.196		7.097	
158	2,3,3′,4,4′,6-Hexachlorobiphenyl		6.397		6.630	
159	2,3,3′,4,5,5′-Hexachlorobiphenyl		6.830		7.001	
160	2,3,3′,4,5,6-Hexachlorobiphenyl		6.441		6.589	
161	2,3,3′,4,5′,6-Hexachlorobiphenyl		6.884		6.727	
162	2,3,3′,4′,5,5′-Hexachlorobiphenyl		6.872		6.947	
163	2,3,3′,4′,5,6-Hexachlorobiphenyl		6.489		6.539	
164	2,3,3′,4′,5′,6-Hexachlorobiphenyl		6.589		6.673	
165	2,3,3′,5,5′,6-Hexachlorobiphenyl		6.602		6.646	
166	2,3,4,4′,5,6-Hexachlorobiphenyl		6.736		6.723	
167	2,3′,4,4′,5,5′-Hexachlorobiphenyl		6.775		7.039	
168	2,3′,4,4′,5′,6-Hexachlorobiphenyl		6.726		6.675	
169	3,3′,4,4′,5,5′-Hexachlorobiphenyl		7.262		7.261	
170	2,2′,3,3′,4,4′,5-Heptachlorobiphenyl		7.087		7.148	
171	2,2′,3,3′,4,4′,6-Hepatchlorobiphenyl		6.458		6.763	
172	2,2′,3,3′,4,5,5′-Heptachlorobiphenyl		7.174		7.059	
173	2,2′,3,3′,4,5,6-Heptachlorobiphenyl		6.500		6.722	
174	2,2′,3,3′,4,5,6′-Heptachlorobiphenyl		6.606		6.818	
175	2,2′,3,3′,4,5′,6-Heptachlorobiphenyl		6.566		6.874	
176	2,2′,3,3′,4,6,6′-Heptachlorobiphenyl		6.536		6.630	
177	2,2′,3,3′,4,5′,6′-Heptachlorobiphenyl		6.701		6.840	
178	2,2′,3,3′,5,5′,6-Heptachlorobiphenyl		6.658		6.783	
179	2,2′,3,3′,5,6,6′-Heptachlorobiphenyl		6.567		6.610	
180	2,2′,3,4,4′,5,5′-Heptachlorobiphenyl		7.426		7.242	
181	2,2′,3,4,4′,5,6-Heptachlorobiphenyl		6.786		6.856	
182	2,2′,3,4,4′,5,6′-Heptachlorobiphenyl		6.892		6.824	
183	2,2′,3,4,4′,5′,6-Heptachlorobiphenyl		6.860		7.004	
184	2,2′,3,4,4′,6,6′-Heptachlorobiphenyl		6.667		6.805	
185	2,2′,3,3′,5,5′,6-Heptachlorobiphenyl		6.890		6.631	
186	2,2′,3,4,5,6,6′-Heptachlorobiphenyl		6.790		6.914	
187	2,2′,3,4′,5,5′,6-Heptachlorobiphenyl		6.953		6.501	
188	2,2′,3,4′,5,6,6′-Heptachlorobiphenyl		6.430		7.447	
189	2,3,3′,4,4′,5,5′-Heptachlorobiphenyl		7.241		7.135	
190	2,3,3′,4,4′,5,6-Heptachlorobiphenyl		6.919		7.228	
191	2,3,3′,4,4′,5′,6-Heptachlorobiphenyl		7.251		7.146	
192	2,3,3′,4,5,5′,6-Heptachlorobiphenyl		6.972		7.093	
193	2,3,3′,4′,5,5′,6-Heptachlorobiphenyl		7.016		7.558	
194^1^	2,2′,3,3′,4,4′,5,5′-Octachlorobiphenyl	7.67	7.554	1.51%	7.558	1.46%
195	2,2′,3,3′,4,4′,5,6-Octachlorobiphenyl		7.114		7.287	
196	2,2′,3,3′,4,4′,5,6′-Octachlorobiphenyl		6.985		7.321	
197	2,2′,3,3′,4,4′,6,6′-Octachlorobiphenyl		7.144		7.191	
198	2,2′,3,3′,4,5,5′,6-Octachlorobiphenyl		7.219		7.336	
199	2,2′,3,3′,4,5,5′,6′-Octachlorobiphenyl		7.291		7.267	
200	2,2′,3,3′,4,5,6,6′-Octachlorobiphenyl		7.201		7.177	
201^1^	2,2′,3,3′,4,5′,6,6′-Octachlorobiphenyl	7.21	7.173	0.51%	7.104	1.47%
202	2,2′,3,3′,5,5′,6,6′-Octachlorobiphenyl		6.685		6.928	
203	2,2′,3,4,4′,5,5′,6-Octachlorobiphenyl		7.551		7.465	
204	2,2′,3,4,4′,5,6,6′-Octachlorobiphenyl		7.163		7.084	
205	2,3,3′,4,4′,5,5′,6-Octachlorobiphenyl		7.380		7.593	
206	2,2′,3,3′,4,4′,5,5′,6-Nonachlorobiphenyl		7.566		7.697	
207^1^	2,2′,3,3′,4,4′,5,6,6′-Nonachlorobiphenyl	7.52	7.571	0.68%	7.627	1.42%
208	2,2′,3,3′,4,5,5′,6,6′-Nonachlorobiphenl		7.091		7.375	
209	Decachlorobiphenyl		7.461		7.875	

1 Training set.

2 Test set.

From Table 2, in the CoMFA and CoMSIA model prediction, the relative errors of the test set (3 compounds) were 0.12, 2.20, 6.41% and 2.86, 1.72, 7.31%, respectively, which are acceptable for small base values.

### Substitution sites determination of log*K*_ow_ value PCBs molecular based on 3D-QSAR/HQSAR models

#### PCBs low biological enrichment substitution sites analysis based on HQSAR model

Based on the default setting A/B/C of the fragment distinction, eight kinds of fragment distinction combinations (A/B/C, A/B/C/H, A/B/C/Ch, A/B/C/DA, A/B/C/H/Ch, A/B/C/H/DA, A/B/C/Ch/DA, and A/B/C/H/Ch/DA.) are constituted after adding other fragment distinction parameters. The statistical results of the PLS analyses for the training set using different fragment distinctions in combinations with default fragment size (4–7) and 12 default hologram lengths (53, 59, 61, 71, 83, 97, 151, 199, 257, 307, 353, and 401) were summarized in Table 3.

**Table 3:** HQSAR model for distinguishing parameters of different fragments

Model	Fragment distinction	Fragment size	r^2^	q^2^	SEE	SE_cv_	HL
1	A/B/C		0.895	0.817	0.281	0.371	257
**2**	**A/B/C/H**		**0.905**	**0.835**	**0.255**	**0.336**	**61**
3	A/B/C/Ch		0.895	0.817	0.281	0.371	257
4	A/B/C/DA	4–7	0.895	0.817	0.281	0.371	257
5	A/B/C/H/Ch		0.905	0.835	0.255	0.336	61
6	A/B/C/H/DA		0.905	0.835	0.255	0.336	61
7	A/B/C/Ch/DA		0.895	0.817	0.281	0.371	257
8	A/B/C/H/Ch/DA		0.905	0.835	0.255	0.336	61

Fragment distinction, A-atom types, B-bond types, C-connectivity, H-hydrogens, Ch-chirality, DA-donor and acceptor. Abbreviations: q^2^, squared cross-validated correlation coefficient; r^2^, squared multiple correlation coefficients; SEE, noncross-validated standard error.

The bold indicates the optimal fragment distinction combinations.

It can be seen from Table 3 that each parameter result of fragment distinction combinations among A/B/C and A/B/C/Ch, A/B/C/DA, A/B/C/Ch/DA was exactly the same, and each parameter result was identical for fragment distinction combinations A/B/C/H and A/B/C/H/Ch, A/B/C/H/DA, A/B/C/H/Ch/DA, indicating that the fragment distinction parameter Ch cannot distinguish the fragment, and it will not make any differences in Ch of molecules with the introduction of fragment distinction parameter DA. That is to say, PCBs molecules do not contain chiral atoms, by the same token, the fragment distinction parameter DA cannot distinguish fragments, and the introduction of fragment distinction parameter Ch did not make the statistical results of model differences, that is, there were no hydrogen bond donor atoms and hydrogen bond acceptor atoms in PCB molecules. This result is the same as that of the CoMFA model, confirming each other.

It was found that the r^2^ of above eight fragment distinction combinations were greater than 0.8, and q^2^ were greater than 0.5. Often, a high value of this statistical characteristic (r^2^ > 0.8, q^2^ > 0.5) is considered as a proof of the high predictive ability of the model. Taking into account the predictive SEE and SE_cv_ values, A/B/C/H was proved as the most effective fragment distinction combinations to build the HQSAR model. According to HQSAR contribution maps, we can get the favorable information for molecular modification.

**Figure 2:**
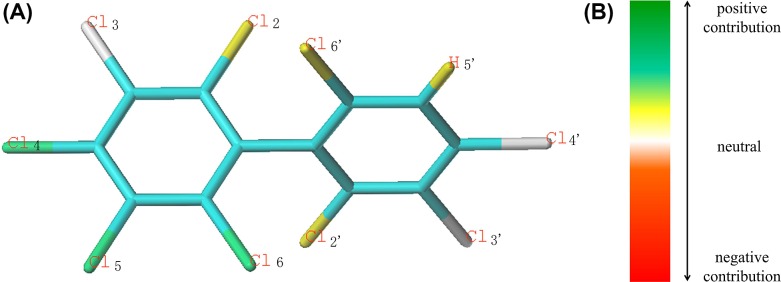
Activity contribution diagram of PCB-207 (**A**), contribution maps of optimal HQSAR model (**B**). Activity contribution diagram of PCB-207 (**A**), contribution maps of optimal HQSAR model (**B**).

As shown in Figure 2B, the order of color about the contribution to activity is: green > green blue > yellow > white > orange > red orange > red. The color from green to red represents the positive contribution (PC) to negative contribution to the activity. As can be seen from Figure 2, the green-labeled Cl_4_, Cl_5_, Cl_6_ molecular Cl-substitutions have a PC to the bioaccumulation activity.

#### CoMFA and CoMSIA 3D contour map analysis

**Figure 3:**
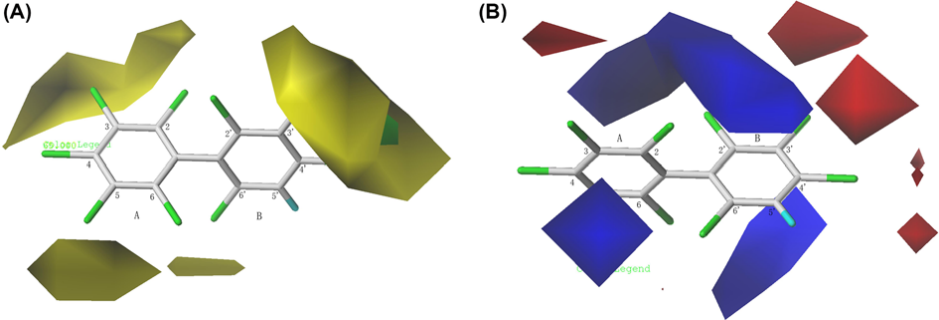
Contour maps of the CoMFA model: (**A**) steric fields and (**B**) electrostatic fields. Contour maps of the CoMFA model: (**A**) steric fields and (**B**) electrostatic fields.

Taking the most active compound PCB-207 as an example, the properties of PCB molecules were analyzed by using 3D contour map of the model. The steric field is denoted by yellow and green colored contours, where the yellow regions represent the small volume groups near these regions favorable to log*K*_ow_, and the green regions indicate that the sterically bulkier substituents close to these regions may increase log*K*_ow_. In the electrostatic field, blue-colored contours represent regions where the positive charge increases the log*K*_ow_ values, whereas red-colored regions display areas where the negative charge enhances the log*K*_ow_ values. Moreover, the yellow and white contours depict hydrophobic and hydrophilic favored regions, respectively. To visualize the field effects on the target compounds in 3D space, the contour diagrams of the final CoMFA and CoMSIA models are shown in Figure 3.

The 3D contour maps of CoMFA model were seen from Figure 3. In steric fields (Figure 3A), large yellow contours located at the 3, 4, 5-positions of the A ring and 3′, 4′-positions of B ring and indicated that the sterically smaller substituent is favored at these positions. A medium-sized region of yellow contour covered the 6′-position of the B ring, indicating that these positions were preferred for smaller substituents to increase log*K*_ow_, that is, increase its solubility in organic phase, reduce its solubility in water, as illustrated by the fact that the log*K*_ow_ value of PCB-207 was stronger than that of compound PCB-201, that is to say, introducing bulky Cl-substituent that at site 5 decreased the solubility of PCBs in the organic phase. Cl_2_, Cl_4_, Cl_5_, Cl_6_, Cl_3_′, Cl_6_′ atoms were located in the blue contours of the electrostatic contour map, and Cl_3_, Cl_3_′, Cl_4_′ atoms were located in the red contours of the electrostatic contour map (Figure 3B), indicating that imbedding electron withdrawing groups at 2, 4, 5, 6, 3′, 6′-positions and imbedding electronegative groups at 3, 3′, 4′-positions can increase the log*K*_ow_ of PCBs. The higher log*K*_ow_ of PCB-194 compared with PCB-207 was an example of such a case.

**Figure 4:**
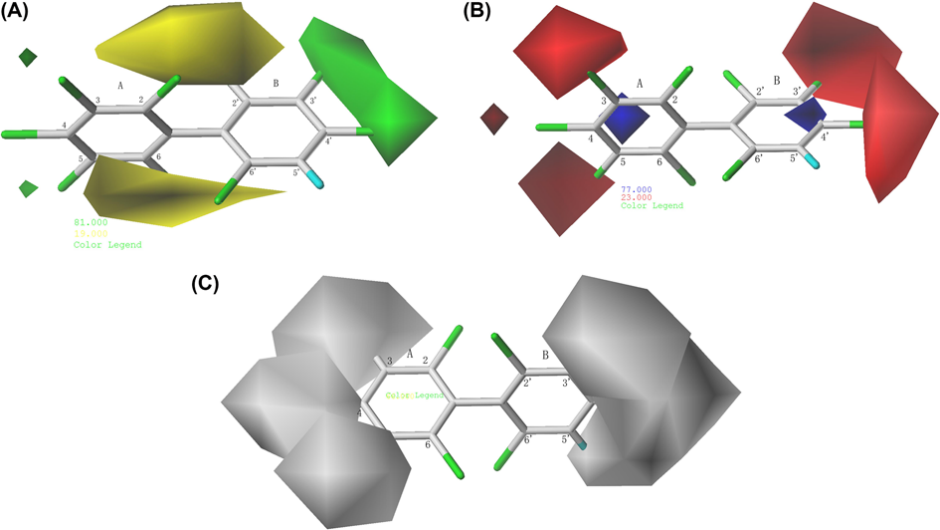
Contour maps of CoMSIA model: (**A**) steric fields, (**B**) electrostatic fields, and (**C**) hydrophobic fields. Contour maps of CoMSIA model: (**A**) steric fields, (**B**) electrostatic fields, and (**C**) hydrophobic fields.

As shown in Figure 4, Cl_2_, Cl_2_′ atoms and carbon skeleton near the Cl_5_, Cl_6_, Cl_6_′ atoms were located in yellow contours of the steric contour map, Cl_3_′, Cl_4_, atoms were located in green contours of the steric contour map, and the small green colored contours were mapped near the 3- and 5-positions, indicating that small volume groups at 2-, 2′-positions and bulkier groups at 3-, 5-, 3′-, 4′-positions may increase log*K*_ow_. In electrostatic fields, two small blue colored contours were mapped the carbon skeleton near the Cl_4_ and Cl_4_′ atoms, the 3-, 4-, 5-, 3′-, and 4′-positions are encompassed by medium-sized region of red contours, representing the electronegative groups at 3-, 4-, 5-, 3′-, 4′-Substitution were favorable to increase log*K*_ow_of PCBs. According to Figure 4C, two large white contour located at the 3-, 4-, 5-positions and 3′-, 4′-, 5′-positions, respectively, indicated that the sterically bulkier substituent is favored at above positions to increase the log*K*_ow_ of PCBs. From the contribution rates of the descriptor fields, the two models mutually verify and prove that electronic effects primarily influence the log*K*_ow_.

Although the models exhibited a satisfactory fitting ability, stability, and predictive ability, only the CoMFA model contained two descriptor fields (steric and electrostatic fields), presenting certain limitations in terms of the analysis of the effect of the descriptor field and the modification of the information on the compounds. The CoMSIA model contained five descriptor fields (steric fields, electrostatic fields, hydrophobic fields, and hydrogen bond donor/acceptor fields), which provided a more comprehensive understanding of the effect on the physical and chemical properties of PCBs. For example, an analysis of the hydrophobic field could reveal that hydrophobic groups on the 3-, 4-, 5-, 3′-, 4′-, and 5′-positions of the benzene ring would increase the log*K*_ow_.

Furthermore, by comparing 3D contour maps of the CoMFA and CoMSIA models, we found that massive regions of the CoMFA contour maps were larger, and the distribution of molecular fields was wide, making it difficult to pinpoint the influence area. Therefore, contour maps of the CoMFA model were selected to modify compounds.

### Molecule modification of low biological enrichment newly designed PCB-207 molecules based on 3D-QSAR/HQSAR models

The effect of each field on the substitution characteristics of each site of the target molecular can be obtained by 3D-QSAR model, and the HQSAR model can determine the substitution sites that contribute most to the bioaccumulation of the compounds. Combined with the 3D contour plot of 3D-QSAR CoMSIA model and activity contribution diagram of HQSAR model, the target molecular can be modified to the low biological enrichment newly designed PCBs-207 molecules by determining the substitution sites and substituent groups.

As can be known from target molecular activity contribution diagram of HQSAR, Cl_4_, Cl_5_, Cl_6_ molecular Cl-substitutions have an uppermost PC to the bioaccumulation activity. A synthetic analysis of the effect of steric, electrostatic, and hydrophobic fields in the CoMSIA model showed that Cl_5_ molecular Cl-Substitution was located in green contours of the steric contour map; Cl_4_, Cl_5_ atoms were located in the red contours of the electrostatic contour map; Cl_4_, Cl_5_ atoms were located in the white contours of the hydrophobic contour map. Know then, Cl_4_ molecular Cl-Substitution was affected by the electrostatic fields and hydrophobic fields, Cl_5_ atom was affected by three molecular fields of the steric fields, electrostatic fields and hydrophobic fields. According to the PLS analysis in Table 1, the contribution rate of the steric fields under the CoMSIA model is only 0.90%, so only the influence of the electrostatic fields and the hydrophobic fields on the molecular modification is considered. Therefore, it was conducive to reducing the *K*_ow_ value of monosubstituted compounds introducing electron withdrawing or hydrophobic groups at Cl_4_, Cl_5_ two positions, respectively, and reducing the *K*_ow_ value of the bis-substituted compounds introducing electron withdrawing or hydrophobic groups at Cl_4_, Cl_5_ positions at same time.

Based on the above analysis, ten kinds of groups with less electronegativity than Cl atom and hydrophobic were introduced to modify the target molecular (PCB-207) at Cl_4_ and Cl_5_ Cl-substitutions. A total of 63 monosubstituted and bis-substituted substitution schemes were established by combining substitution sites and substituent groups. According to the Predicted log*K*_ow_ values of newly designed PCB-207 molecules through CoMFA and CoMSIA models, the *K*_ow_ values of newly designed PCB-207 compounds decreased by embedding the groups with less electronegativity than Cl atom (-OH, -CH_2_OH, -NH_2_, -NO), and groups hydrophobic besides less electronegativity (-CH_2_CH_3_, -Br, -CH_3_, -NO_2_, -Phenyl, -OCH_3_), indicating that the introduction of groups is the reason for the descending *K*_ow_ values, the other words descending biological enrichment of the modified compounds.

### POPs characteristic evaluation of low biological enrichment newly designed PCB-207 molecules

From the above analysis, it is known that the bioaccumulation of newly designed PCB-207 molecules is reduced. However, in addition to the bioaccumulation of the four POPs characteristics, the toxicity, persistence, and long-range mobility of the low biological enrichment newly designed PCB-207 molecules needs to be evaluated and analyzed. A total of 32 low biological enrichment newly designed PCB-207 molecules with a *K*_ow_ value reduced by more than 10% were selected from 63 newly designed PCB-207 molecules mentioned above and evaluated for toxicity, persistence and long-range mobility to further screen out the optimally modified compounds, and evaluate its functional properties that is to say the flame retardancy, insulation evaluation.

Li et al. [29] constructed the 3D-QSAR model to predict the toxicity parameter *pEC_50_* of PCBs, Xu et al. [30] constructed the 3D-QSAR model to predict the persistence parameter t_1/2_ of PCBs, and Chen et al. [31] constructed the 3D-QSAR model to predict the long-distance mobility parameter *K*_OA_ of PCBs, the present paper will use above model to predict the toxicity, long-lasting, and long-distance mobility of newly designed PCB-207 molecules.

Based on the analysis of 32 new molecules, we knew that the log*K*_ow_ values of 32 newly designed PCB-207 molecules decreased significantly compared with PCB-194 and PCB-201, indicating that the introduction of groups is the reason for the descending *K*_ow_ values of the modified compounds and not the decrease in Cl. Thirty-two kinds of low biological enrichment newly designed PCB-207 molecules with a log*K*_ow_ value reduced by more than 10% are mostly obtained after being modified and substituted at two substitution sites at same time, thus the log*K*_ow_ reduction in bis-substituted newly designed molecules is greater than that of monosubstituted newly designed compounds. 12-low biological enrichment newly designed PCB-207 molecules with reduced toxicity, persistence, and long-distance mobility were screened from 32 compounds, and these 12 newly designed PCB-207 molecules were calculated using Gaussian09 for their functional properties. (63 substitution schemes of new designed PCB-207 molecules are shown in the supplementary material.)

### Functional properties (flame retardancy, insulativity) evaluation of low biological enrichment newly designed PCB-207 molecules

Energy gap (insulativity parameter) and C–Cl bond dissociation enthalpy (flame retardancy parameter) of PCB-207 molecules and low biological enrichment newly designed PCB-207 molecules were calculated using Gaussian09 showed in Table 4. The insulation information of newly designed molecules can be obtained by calculating the energy gap value, in the same way, the flame retardancy information about low biological enrichment newly designed PCB-207 molecules can be obtained by calculating the C–Cl bond dissociation enthalpy.

**Table 4:** Structural modification of newly designed PCB-207 molecules, the predicted values of log*K*_ow_, logK_OA_, logt_1/2_, and *pEC50* by CoMFA and CoMSIA models and calculated values of energy gap and C–Cl bond dissociation enthalpy by Gaussian

Compounds	Predicted log*K*_ow_		Predicted log*K_OA_*		Predicted log_t1/2_		Predicted *pEC50*		Energy gap	Change in rate of energy gap (%)	C–Cl bond dissociation enthalpy (kcal/mol)
	CoMFA	CoMSIA	CoMFA	CoMSIA	CoMFA	CoMSIA	CoMFA	CoMSIA			
PCB-207	10.613	11.538	10.613	11.538		1.889	5.672	5.646	0.20367		70.706
5-Methyl-PCB-207	6.124	6.781	9.799	10.777	1.198	1.474	4.496	4.873	0.20641	1.35%	24.698
5-Amino-PCB-207	6.241	6.849	9.245	10.681	1.255	1.386	4.091	3.987	0.19920	−2.19%	25.405
4-Oxhydryl-5-methyl-PCB-207	6.738	6.850	10.407	10.649	1.404	1.454	4.182	4.555	0.19884	−2.37%	25.624
4-Hydroxymethyl-5-methyl-PCB-207	6.111	6.404	9.055	10.015	1.109	1.388	4.003	3.657	0.20253	−0.56%	25.033
4-Amino-5-ethyl-PCB-207	6.748	6.488	9.995	9.918	1.299	1.429	3.115	2.869	0.20327	−0.20%	25.279
4-Amino-5-phenyl-PCB-207	6.483	6.907	10.196	10.924	1.148	1.543	4.438	4.670	0.20140	−1.11%	25.371
4-Bromine-5-hydroxymethyl-PCB-207	6.017	6.813	8.726	10.481	1.052	1.365	4.539	5.198	0.20449	0.40%	27.039
4-Ethyl-5-hydroxymethyl-PCB-207	6.374	6.471	10.425	10.361	1.283	1.510	3.322	4.360	0.20475	0.53%	25.212
4-Methyl-5-hydroxymethyl-PCB-207	6.288	6.395	9.927	10.086	1.278	1.316	5.108	4.792	0.20477	0.54%	25.021
4-Phenyl-5-hydroxymethyl-PCB-207	6.078	6.796	8.601	8.165	1.041	1.324	4.437	4.945	0.20076	−1.43%	24.872
4-Phenyl-5-amino-PCB-207	6.146	6.741	8.683	8.309	1.160	1.404	3.924	4.049	0.20237	−0.64%	25.887
4-Methyl-5-methyl-PCB-207	5.898	6.236	9.809	10.002	1.118	1.424	4.184	4.340	0.20283	−0.41%	25.021

As shown in Table 4, the dissociation enthalpies of C–Cl bonds are lower than those of the target molecular PCB-207 (70.706 kcal/mol) under the condition that four POPs characteristic parameters of 12 low biological enrichment newly designed PCB-207 molecules are reduced. Studies have shown that PCBs will release HCl during combustion process, capturing and reacting with highly active H and OH radicals produced during the combustion reaction of polymer materials sequentially, eventually leading to the slowdown or termination of combustion [32,33]. The dissociation of C–Cl bond is closely related to the flame retardant efficiency of PCBs, indicating that the low biological enrichment newly designed PCB-207 molecules after being modified are more likely to release Cl-free radicals and HCl for inflaming retarding. The energy gap values increase in four low biological enrichment new designed PCB-207 molecules (5-methyl-PCB-207, 4-bromine-5-hydroxymethyl-PCB-207, 4-ethyl-5-hydroxymethyl-PCB-207, and 4-methyl-5-hydroxymethyl-PCB-207) was 0.40–1.35%, indicating that the insulation increased; and the energy gap values decreased of the rest eight low biological enrichment newly designed PCB-207 molecules was from −0.37 to 0.20%, the amplitude of increase or decrease both are small, thus explaining that the effect on the degree of energy gap of low biological enrichment modified PCB-207 compounds improves the flame retardancy while the insulation remained essentially unchanged.

### Mechanism analysis of biological enrichment of PCBs

#### Analysis of the effects of Cl substitutes on bioaccumulation of PCBs

There are ten Cl isomers of PCBs, one to ten chlorinated biphenyls. The values of 209 kinds of PCBs molecules in Table 2 are used for predicting the mean value of log*K*_ow_ of PCBs from one to ten chlorine number. The values of fitting correlation coefficients R^2^ for the value of log*K*_ow_ and the number of chlorine atom under CoMFA and CoMSIA model are 0.70911 and 0.81723 (*n*=10, *P*=0.01, r > r_0_), respectively. The values of R^2^ for the mean value of log*K*_ow_ and the number of chlorine atom under each category are 0.9907 and 0.9992 (*n*=209, *P*=0.01, r > r_0_), respectively. These results show that the values of log*K*_ow_ gradually increased with the increasing number of Cl atoms, and stronger *K*_ow_ values of PCBs imply a stronger biological enrichment ability of PCBs. Thus, the biological enrichment increased with the increasing number of Cl atoms.

The names of the five most common PCB mixtures commodity are Aroclor1016 (41.5% Cl), Aroclor1242 (42% Cl), Aroclor1248 (48% Cl), Aroclor1254 (54% Cl), and Aroclor1260 (60% Cl), respectively. Except for Aroclor1016, the first two digits of other commodity name are 12, which represent the number of carbon atoms in PCB molecules. The last two digits represent the isomer ratios of Cl in the commercial mixtures of PCBs, indicating the name of commodity according to the different degree of chloride.

Table 5 [31] presents the ratio of the isomers contained in each PCBs commodity mixtures, which show that the Cl isomers from trichlorodiphenyl to heptachlorobiphenyl make up most of the commercial products of PCBs, and chlorobiphenyl and dichlorobiphenyl also account for a certain percentage of the total products, while the Cl range from octachlorobiphenyl to decachlorobiphenyl is rarely used in commercial mixture of PCBs.

**Table 5:** The isomer ratios of Cl in the common mixture PCB goods [33]

The Cl isomer	The common PCB mixtures				
	Aroclor1016	Aroclor1242	Aroclor1248	Aroclor1254	Aroclor1260
Chlorobiphenyl	0.7	0.8	0.0	0.0	0.0
Dichlorobiphenyl	17.5	15.0	0.4	0.2	0.1
Trichlorobiphenyl	54.7	44.9	22.0	1.3	0.2
Tetrachlorobiphenyl	26.6	32.6	56.6	16.4	0.5
Pentachlorobiphenyl	0.5	6.4	18.6	53.0	8.6
Hexachlorobiphenyl	0.0	0.3	2.0	26.8	43.4
Heptachlorobiphenyl	0.0	0.0	0.6	2.7	38.5
Octachlorobiphenyl	0.0	0.0	0.0	0.0	8.3
Nonachlorobiphenyl	0.0	0.0	0.0	0.0	0.7
Decachlorobiphenyl	0.0	0.0	0.0	0.0	0.0

The following PCB congeners were detected in *Lampetra fluviatilis* in Europe by Merivirta et al. [34]: PCB-28, 52, 101, 114, 118, 128, 138, 141, 149, 151, 153, 170, 180. The range of these congeners is from trichlorodiphenyl to heptachlorobiphenyl. The study of PCB accumulation and dietary exposure to fish in Hyderabad, India, by Ahmed et al. [35], found that PCB congeners in all fish species are distributed in the dichlorobiphenyl to decachlorobiphenyl. Russo et al. [36] traced PCBs enriched in water samples found that PCB homologs recovered from water samples contained (PCB-1, 15, 31, 44, 138, 180, 195), in which the range is chlorobiphenyl, dichlorobiphenyl, trichlorodiphenyl, tetrachlorobiphenyl, hexachlorobiphenyl, heptachlorobiphenyl, and octachlorobiphenyl. This is consistent with the finding in Table 5 that the range of almost all PCBs that can be detected in all aquatic organisms cover trichlorodiphenyl to heptachlorobiphenyl. The results showed that the more the certain PCBs homolog used, the more the detection.

#### Mechanism analysis of biological enrichment of PCBs based on molecular docking

Take 209 kinds of PCBs bioaccumulation in fish as the target molecule docking with its degrading enzyme, getting the corresponding scoring function associated with its bioaccumulation. The higher the scoring function, the better the PCBs and degrading enzymes bind, and the easier degradable PCB molecules are, the lower the bioaccumulation is. Therefore, the relationship between the number of Cl atoms and the bioaccumulation is verified by this way. BphA is the only enzyme direct contact with PCBs in biodegradation. Thus, BphA was used to dock with 209 PCBs using protein structure of 2GBX [37].

According to the scoring functions of all the Cl isomers from chlorobiphenyl to decachlorobiphenyl binding with BphA enzyme, the average Total Score under each Cl atom classification was 3.087, 2.564, 2.187, 1.560, 1.177, 0.817, 0.633, 0.432, 0.427, and 0.130, respectively, the average Total Score under each Cl atom classification of PCBs binding with BphA enzyme and the number of Cl atoms fit the curve y= −0.32417x + 3.08427, R^2^ = 0.93251, thus r = 0.9656. When the significance level *P*=0.01, r_0_ = 0.7645, there was a significant linear relationship between the total score of PCBs binding to BphA enzyme and the number of Cl atoms in each classification (*n*=10, *P*=0.01, r > r_0_). The higher the total score, the better the binding of PCB molecules with the BphA enzyme, and the lower the biological enrichment. As the number of Cl atoms increases, the total score shows a decreasing trend, which indicating that highly chlorinated PCBs are not easily degraded and more easily enriched in the fish body, that is, the number of Cl atoms increases, their biological enrichment follows enhanced. In addition, a linear correlation between the total score obtained by molecular docking between 209 PCBs and BphA enzyme and the number of corresponding Cl atoms was obtained, the correlation coefficient r^2^ was 0.6757.

It can be seen from Table 6 that when the significance level *P*=0.01, the r of the four linear fitting relationships is greater than the correlation coefficient test critical value r_0_, indicating that the log*K*_ow_ values of PCB molecules and the total scores of docking with BphA enzymes (represent biological enrichment) and the number of Cl atoms have a strong correlation.

**Table 6:** Correlation coefficient critical value test of PCBs predicted log*K*_ow_ values and total scores with number of chlorines

	*P*	*n*	r_0_	r	
				CoMFA	CoMSIA
The linear fitting of Cl atoms compared with 10 log*K*_ow_ average values	0.01	10	0.76459	0.99070	0.99920
The linear fitting of Cl atoms compared with 209 log*K*_ow_ values	0.01	209	0.10898	0.84219	0.90401
The linear fitting of Cl atoms compared with 10 average values of Total Scores	0.01	10	0.76459	0.96567	
The linear fitting of Cl atoms compared with 209 PCBs Total Scores	0.01	209	0.10898	0.82205	

The log*K*_ow_ values used for modeling comes from experimental determinations and belongs to the property values of PCB molecules, but the effect of bioaccumulation on specific receptor proteins is unknown. In order to further investigate the effect of low biological enrichment newly designed PCBs-207 molecules on the bioaccumulation of liver enzymes in fish, 12 newly designed PCB-207 molecules, in which all the POPs characteristic parameters are reduced while the actual functional properties are not changed were respectively docked with aryl hydrocarbon receptor (AHR) [38], superoxide dismutase (SOD) [39], cytochrome P450-dependent monooxygenases (CYP1A) [40], ethoxyresorufin-o-deethylase (EROD) [41], glutathione s-transferase (GST) [42], catalase (CAT) [43], and the aryl hydrocarbon hydroxylase (AHH) [44] to verify whether the 12 newly designed PCB-207 molecules have low bioaccumulation for various enzymes. The structures of seven receptor proteins are all from the Protein Data Bank (http://www.rcsb.org/pdb), SOD is an antioxidant enzyme located in erythrocytes of the liver, which the enzymatic activity reduced due to PCBs entering [45]. EROD enzyme was shown to directly contact PCBs and is regulated by CYP1A enzymes. The higher the concentration of pollutants entering the fish, the more the enzyme quantity, and the higher the enzyme activity [46]. When the pollutants enter the body of the tested zebrafish, the GST in liver activity decreases [47].

**Figure 5:**
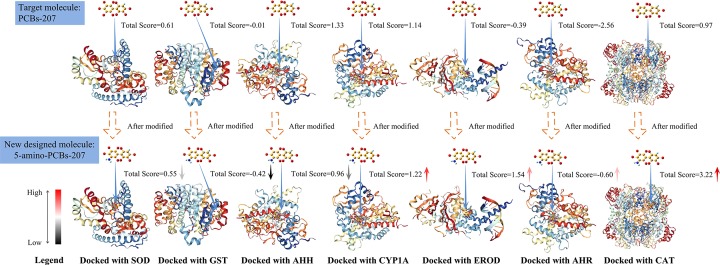
Schematic diagram of PCB-207 and newly designed PCB-207 (take 5-amino-PCB-207 as an example) docking with seven enzymes and change of total scores

As shown in Figure 5, taking the modified molecule 5-amino-PCB-207 as an example, compared with the target molecule PCB-207, the total score of 5-amino-PCB-207 molecular docking with SOD, GST, and AHH had declined. According to the color of the legend, the decreasing order of total score values for 5-amino-PCB-207 docking with enzymes is GST > AHH > SOD; the total score of 5-amino-PCB-207 molecular docking with CYP1A, EROD, AHR, and CAT had increased. According to the color of the legend, the increasing order of total score values for 5-amino-PCB-207 docking with enzymes is CAT > CYP1A > EROD > AHR.

The decrease in total score indicated that the binding of 5-amino-PCB-207 with SOD, GST, and AHH had got worse, which could reduce PCBs’ enrichment in liver. Although the increase in total score indicated that the degree of biological enrichment for 5-amino-PCB-207 in CYP1A, EROD, AHR, and CAT did not decrease, that is, not all of the 12 low biological enrichment new designed PCB-207 molecules reduce the bioaccumulation at liver enzymes, the present paper only provides a method to study the bioaccumulation effects of new molecules on specific receptors by using molecular docking.

Table 7 showed the total scores of 12 low biological enrichment newly designed PCB-207 molecules after docking with seven enzymes. It can be seen from the total scores in Table 7, 5-methyl-PCB-207, 5-amino-PCB-207 and 4-amino-5-ethyl-PCB-207, the binding extent of each newly designed molecular mentioned above to the three enzymes was decreasing compared with that of the target molecular PCB-207. For example, the total scores of 5-methyl-PCB-207 docking with SOD, GST and CAT enzymes were lower than the total scores of the target molecular PCB-207 docking with the above three enzymes; the total scores of 5-amino-PCB-207 docking with SOD, GST, and AHH enzymes were lower than the total scores of the target molecular PCB-207 docking with the above three enzymes; the total scores of 4-amino-5-ethyl-PCB-207 docking with EROD, GST, and AHH enzymes were lower than the total scores of the target molecular PCB-207 docking with the above three enzymes.

**Table 7:** Total scores of PCB-207 and new designed PCB-207 docking with seven enzymes

Compounds	Total score						
	SOD	CYP1A	EROD	GST	AHR	AHH	CAT
PCB-207	0.61	−2.56	1.14	−0.01	−0.39	1.33	0.97
5-methyl-PCB-207	**0.56**	0.22	1.65	**−0.2**	1.03	1.42	**−0.45**
5-amino-PCB-207	**0.55**	−0.6	1.22	−**0.42**	1.54	**0.96**	3.22
4-oxhydryl-5-methyl-PCB-207	**0.57**	−0.19	2.33	−**0.32**	2.28	1.92	3.22
4-hydroxymethyl-5-methyl-PCB-207	2.43	0.48	2.15	0.85	2.35	1.55	2.84
4-amino-5-ethyl-PCB-207	1.73	0.55	**0.80**	**−0.18**	1.18	**1.29**	1.42
4-amino-5-phenyl-PCB-207	2.30	−2.28	2.93	0.90	1.07	**1.32**	3.92
4-bromine-5-hydroxymethyl-PCB-207	**0.53**	1.00	1.17	0.08	0.85	**1.22**	3.04
4-ethyl-5-hydroxymethyl-PCB-207	**0.52**	−0.22	2.92	0.54	2.80	1.39	4.86
4-methyl-5-hydroxymethyl-PCB-207	**0.50**	−0.89	1.34	1.00	2.94	**1.21**	1.31
4-phenyl-5-hydroxymethyl-PCB-207	1.37	−1.63	1.68	1.86	4.00	2.38	4.20
4-phenyl-5-amino-PCB-207	0.87	−0.57	**0.72**	0.24	1.04	2.13	4.03
4-methyl-5-methyl-PCB-207	**0.44**	0.93	1.42	0.54	2.21	1.56	3.18

The bold indicates the total scores of new designed PCB-207 docking with emzymes that were lower than the Total scores of that of PCB-207.

## Conclusion

1. In the present study, HQSAR model was constructed to determine that the Cl-substitutions Cl_4_, Cl_5_, and Cl_6_ have an uppermost PC to the bioaccumulation activity. Based on 3D contour plot of CoMSIA model, indicated that introducing electron withdrawing or hydrophobic groups into Cl_4_ and Cl_5_ substitution sites of PCB molecules could decrease the bioaccumulation of target molecular. Coupled with HQSAR and CoMSIA model, reduced the *K*_ow_ value of monosubstituted compounds by introducing electron withdrawing or hydrophobic groups at Cl_4_, Cl_5_ two positions, respectively, and reducing the *K*_ow_ value of the bis-substituted compounds by introducing electron withdrawing or hydrophobic groups at Cl_4_, Cl_5_ positions at same time.
2. Based on HQSAR/CoMSIA model, 12 kinds of novel PCB-207 molecules were designed, which have lower biological enrichment, toxicity, persistence, and long-distance migration ability while the actual functional properties (insulation and flame retardancy) are not changed. Based on the molecular docking, the bioaccumulation mechanism of PCBs shows that the biological enrichment effect of highly chlorinated PCBs is significantly higher than that of PCBs with less Cl substituents. Through the molecular docking, it can also reflect the biological enrichment effect of low biological enrichment new designed PCB-207 molecules on the specific enzymes in the liver of fish and further screen out the environmentally-friendly features of the more obvious low biological enrichment new designed PCB-207 molecules.

## Supporting information

**Supplementary Material:** 

## Abbreviations

AHH: aryl hydrocarbon hydroxylase
AHR: aryl hydrocarbon receptor
CAT: catalase
Ch: chirality
CoMFA: comparative molecular field analysis
CoMSIA: comparative molecular similarity indices analysis
CYP1A: cytochrome P450-dependent monooxygenase
EROD: ethoxyresorufin-o-deethylase
GST: glutathione s-transferase
HOMO: highest occupied molecular orbital
HQSAR: hologram quantitative structure–activity relationship
PCB: polychlorinated biphenyl
PLS: partial least square
POP: persistent organic pollutant
SE_cv_: cross-validated standard error of estimate
SEE: standard error of estimate
SOD: superoxide dismutase
