# Supplementary material for: Biological enrichment prediction of polychlorinated biphenyls and novel molecular design based on 3D-QSAR/HQSAR associated with molecule docking
Source: Biosci Rep. 2019 May 17;39(5):BSR20180409. doi: 10.1042/BSR20180409 (PMC6522710; doi:10.1042/BSR20180409)
Supplement: Supplementary file 1 [file bsr20180409_Supp1.pdf]

Coupled with 3D-QSAR and HQSAR models, 10 kinds of groups with less electronegativity than Cl atom and hydrophobic were introduced to modify the target molecular (PCB-207) at Cl_4 and Cl_5 Cl-substitutions. 63 mono-substituted and bis-substituted substitution schemes were established by combining substitution sites and substituent groups, and the 63 substitution schemes were showed in the following Tables.

Table 1 Predicted logK_{ow} values of new designed PCB-207 molecule through CoMFA and CoMSIA models

Substituted sites	Compounds	Predicted values of logK_{ow}	
		CoMFA	CoMSIA
	PCB-207	7.571	7.627
Mono-substituted in 4-site	4-bromine-PCB-207	7.309	7.500
	4-methoxy-PCB-207	6.773	7.382
	4-ethyl-PCB-207	6.907	6.987
	4-methyl-PCB-207	6.811	6.944
	4-oxhydryl-PCB-207	6.486	7.260
	4-hydroxymethyl-PCB-207	6.806	6.997
	4-amino-PCB-207	6.987	6.954
	4-nitryl-PCB-207	7.160	7.295
	4-phenyl-PCB-207	7.275	7.482
Mono-substituted in 5-site	5-methoxy-PCB-207	7.415	7.154
	5-ethyl-PCB-207	7.234	7.135

	5-methyl-PCB-207	6.124	6.781
	5-oxhydryl-PCB-207	6.960	7.478
	5-hydroxymethyl-PCB-207	6.889	7.091
	5-amino-PCB-207	6.241	6.849
	5-nitryl-PCB-207	7.311	7.321
	5-phenyl-PCB-207	7.121	7.306
Bis-substituted in 4,5-site	4-methoxy-5-bromine-PCB-207	6.770	7.326
	4-methoxy-5-ethyl-PCB-207	6.147	6.785
	4-methoxy-5-methyl-PCB-207	6.063	6.712
	4-methoxy-5-phenyl-PCB-207	5.675	5.843
	4-oxhydryl-5-bromine-PCB-207	6.496	7.197
	4-oxhydryl-5-ethyl-PCB-207	6.823	6.879
	4-oxhydryl-5-methyl-PCB-207	6.738	6.850
	4-oxhydryl-5-nitryl-PCB-207	7.325	7.235
	4-oxhydryl-5-phenyl-PCB-207	6.577	7.173
	4-hydroxymethyl-5-bromine-PCB-207	6.255	6.835
	4-hydroxymethyl-5-ethyl-PCB-207	5.975	6.407
	4-hydroxymethyl-5-methyl-PCB-207	6.111	6.404
	4-hydroxymethyl-5-nitryl-PCB-207	6.636	7.012
	4-hydroxymethyl-5-phenyl-PCB-207	6.113	5.936
	4-amino-5-bromine-PCB-207	6.609	6.934

4-amino-5-ethyl-PCB-207	6.748	6.488
4-amino-5-methyl-PCB-207	5.946	6.343
4-amino-5-nitryl-PCB-207	6.87	6.839
4-amino-5-phenyl-PCB-207	6.483	6.907
4-nitroso-5-methyl-PCB-207	6.955	7.515
4-bromine-5-methoxy-PCB-207	6.891	7.298
4-bromine-5-oxhydryl-PCB-207	6.645	7.293
4-bromine-5-hydroxymethyl-PCB-207	6.017	6.813
4-ethyl-5-methoxy-PCB-207	6.552	6.855
4-ethyl-5-oxhydryl-PCB-207	7.081	7.018
4-ethyl-5-hydroxymethyl-PCB-207	6.374	6.471
4-ethyl-5-amino-PCB-207	6.352	6.501
4-ethyl-5-nitroso-PCB-207	6.973	7.434
4-methyl-5-methoxy-PCB-207	6.831	6.855
4-methyl-5-oxhydryl-PCB-207	6.238	6.801
4-methyl-5-hydroxymethyl-PCB-207	6.288	6.395
4-methyl-5-amino-PCB-207	5.627	6.234
4-methyl-5-nitroso-PCB-207	6.752	7.344
4-nitryl-5-oxhydryl-PCB-207	7.084	7.321
4-nitryl-5-hydroxymethyl-PCB-207	6.278	6.645
4-nitryl-5-amino-PCB-207	7.289	6.998

4-phenyl-5-methoxy-PCB-207	6.888	7.297
4-phenyl-5-oxhydryl-PCB-207	6.652	7.308
4-phenyl-5-hydroxymethyl-PCB-207	6.078	6.796
4-phenyl-5-amino-PCB-207	6.146	6.741
4-phenyl-5-nitroso-PCB-207	5.489	5.973
4-bromine-5-bromine-PCB-207	7.275	7.444
4-ethyl-5-ethyl-PCB-207	6.019	6.282
4-methyl-5-methyl-PCB-207	5.898	6.236
4-nitryl-5-nitryl-PCB-207	7.505	7.178
4-phenyl-5-phenyl-PCB-207	7.101	7.234

A total of 32 low biological enrichment new designed PCB-207 molecules with a Kow value reduced by more than 10% were selected from 63 new designed PCB-207 molecules mentioned above and evaluated for toxicity, persistence and long-range mobility to further screen out the optimal modified compounds. According to the 3D-QSAR constructed by Li, Xu and Chen to predict the toxicity parameter pEC_{50}, persistence parameter $t_{1/2}$ and long-distance mobility parameter K_{OA} of new designed molecules, respectively, the predicting results were shown in Table 2.

Table 2 Migration, persistence and toxicity assessment of new designed PCB-207 molecules

Compounds	Predicted $\log K_{OA}$		Predicted $\log t_{1/2}$		Predicted pEC_{50}	
	CoMFA	CoMSIA	CoMFA	CoMSIA	CoMFA	CoMSIA
PCB-207	10.613	11.538		1.889	5.672	5.646

5-methyl-PCB-207	9.799	10.777	1.198	1.474	4.496	4.873
5-amino-PCB-207	9.245	10.681	1.255	1.386	4.091	3.987
4-methoxy-5-ethyl-PCB-207	10.414	10.583	1.224	1.374	6.263	5.773
4-methoxy-5-methyl-PCB-207	10.315	10.528	1.221	1.36	6.554	5.902
4-oxhydryl-5-methyl-PCB-207	10.407	10.649	1.404	1.454	4.182	4.555
4-hydroxymethyl-5-bromine-PCB-207	10.377	11.027	1.337	1.596	5.236	5.815
4-hydroxymethyl-5-ethyl-PCB-207	9.628	10.278	1.043	1.454	7.693	4.906
4-hydroxymethyl-5-methyl-PCB-207	9.055	10.015	1.109	1.388	4.003	3.657
4-amino-5-bromine-PCB-207	9.805	10.717	1.188	1.525	7.075	5.942
4-amino-5-ethyl-PCB-207	9.995	9.918	1.299	1.429	3.115	2.869
4-amino-5-methyl-PCB-207	9.491	9.798	1.074	1.336	7.667	6.725
4-amino-5-phenyl-PCB-207	10.196	10.924	1.148	1.543	4.438	4.67
4-bromine-5-hydroxymethyl-PCB-207	8.726	10.481	1.052	1.365	4.539	5.198
4-ethyl-5-hydroxymethyl-PCB-207	10.425	10.361	1.283	1.51	3.322	4.36
4-ethyl-5-amino-PCB-207	10.22	10.204	1.351	1.409	5.333	5.754
4-methyl-5-hydroxymethyl-PCB-207	9.927	10.086	1.278	1.316	5.108	4.792
4-methyl-5-amino-PCB-207	9.646	10.084	1.097	1.386	7.187	5.547
4-nitryl-5-hydroxymethyl-PCB-207	9.592	10.705	1.273	1.407	6.073	7.787
4-phenyl-5-hydroxymethyl-PCB-207	8.601	8.165	1.041	1.324	4.437	4.945
4-phenyl-5-amino-PCB-207	8.683	8.309	1.16	1.404	3.924	4.049
4-ethyl-5-ethyl-PCB-207	9.960	10.109	1.051	1.425	7.335	6.091

4-methyl-5-methyl-PCB-207	9.809	10.002	1.118	1.424	4.184	4.34
4-methoxy-5-phenyl-PCB-207	11.360	11.554	1.369	0.913	6.261	6.372
4-oxhydryl-5-bromine-PCB-207	10.730	11.469	1.431	1.633	6.053	5.873
4-oxhydryl-5-phenyl-PCB-207	10.913	11.493	1.439	1.691	5.826	6.828
4-hydroxymethyl-5-nitryl-PCB-207	10.669	11.198	1.415	1.412	5.035	4.084
4-hydroxymethyl-5-phenyl-PCB-207	10.683	11.086	1.354	1.179	5.19	6.22
4-bromine-5-oxhydryl-PCB-207	10.692	11.757	1.337	1.591	5.805	4.887
4-oxhydryl-PCB-207	10.568	11.586	1.23	1.588	6.543	5.985
4-ethyl-5-methoxy-PCB-207	10.690	10.864	1.439	1.519	4.184	4.34
4-methyl-5-oxhydryl-PCB-207	9.974	10.907	1.483	1.516	6.317	4.951
4-phenyl-5-nitroso-PCB-207	11.713	12.748	1.666	1.349	6.125	5.795
